# Epigenetic Mechanisms in the Pathophysiology and Progression of Epilepsy: A Comprehensive Review of Experimental and Clinical Studies

**DOI:** 10.2174/1570159X23666241220163832

**Published:** 2025-02-19

**Authors:** Yinchao Li, Zhengwei Su, Ke Zhao, Xianyue Liu, Shuda Chen, Xiaofeng Yang, Liemin Zhou

**Affiliations:** 1 Department of Neurology, The Seven Affiliated Hospital, Sun Yat-Sen University, Shenzhen, Guangdong Province, 518107, China;; 2 Guangzhou Laboratory, Guangzhou 510005, Guangdong Province, China;; 3 The First Affiliated Hospital, Guangzhou Medical University, Guangzhou 510005, Guangdong Province, China

**Keywords:** Epilepsy, epigenetic regulation, DNA methylation, histone modification, non-coding RNA, RNA modifications

## Abstract

Epilepsy is a prevalent neurological disorder that presents with a diverse range of clinical manifestations and etiologies influenced by both genetic and environmental factors. However, traditional genetic mechanisms alone are insufficient to fully elucidate the pathogenesis of epilepsy, highlighting the increasing importance of epigenetics in epilepsy research. Several studies have demonstrated that epigenetic mechanism play a pivotal role in the development and progression of epilepsy. This review provides a comprehensive overview of epigenetic regulation and its role in epilepsy. We emphasize the specific role of epigenetic regulation, including DNA methylation, non-coding RNA, and histone modification in the epilepsy. Finally, we discuss the potential applications of epigenetic regulation in the etiology research, drug development, and personalized therapy of epilepsy, along with the technical and theoretical challenges that need to be addressed in epigenetic research. Epigenetic mechanisms have emerged as a promising avenue for understanding the pathogenesis and treatment of epilepsy. However, to thoroughly grasp its potential implications for the clinical management of this disease, a deeper understanding of the role of epigenetics in TLE is essential. Therefore, further research is required to elucidate the specific epigenetic mechanisms involved in epilepsy, their interactions with other disease-related factors, and their potential as therapeutic targets. Such research could ultimately lead to the development of novel epigenetic-based therapies for epilepsy and other related neurological disorders.

## 1 INTRODUCTION

A significant proportion of epilepsy patients, approximately 30%, will eventually progress to a state of chronic drug-resistant epilepsy, indicating a lack of response to currently available anti-seizure medications [1]. Its profound impact on public health and individual lives underscores the importance of comprehensive research. The chronic and recurrent nature of epileptic seizures, coupled with the associated rise in mortality rates and substantial economic burden, have a profound negative impact on the overall quality of life [2]. As such, there is a critical need to identify and develop new therapeutic strategies for the management of this disorder. At present, epilepsy is characterized by heterogeneous clinical presentations and etiologies, and the underlying mechanisms involved in its pathogenesis remain incompletely understood. Epilepsy is often accompanied by aberrant gene expression, which affects the gene networks regulating various pathways, including inflammation, gliosis, synaptic structure, and neuronal function [3]. Although multiple causative genes have been implicated in epilepsy, familial linkage analyses have only identified genes that explain less than 1% of cases [4]. The majority of cases exhibit complex genetic inheritance patterns. In recent years, numerous candidate genes have been identified through deep sequencing and linkage analysis, but their replicability is low, and there is a lack of effective functional validation, making epilepsy genetics research challenging [5]. Epigenetic regulation has emerged as a pivotal focus in understanding the intricate mechanisms underlying epilepsy, contributing significantly to the evolving landscape of epilepsy research [6]. This paradigm shift stems from the realization that the conventional view of epilepsy as purely a result of genetic mutations or structural abnormalities falls short in explaining the diverse etiology and pathophysiology observed in patients [7, 8]. At its core, epigenetics refers to heritable changes in gene expression that occur without alterations to the underlying DNA sequence. The most extensively studied epigenetic mechanisms relevant to epilepsy include DNA methylation [9, 10], histone modifications [11, 12], and non-coding RNA-mediated regulation [13, 14]. These processes collectively orchestrate the intricate dance of gene activation and repression, ultimately shaping the cellular and molecular milieu that underlies epileptogenesis. Epigenetics can regulate gene expression, as well as the regulatory networks and protein translation mechanisms of genes, playing an essential role in maintaining axon regeneration, brain development, and brain function [15, 16].

One of the central aspects highlighting the importance of epigenetic regulation in epilepsy is the dynamic interplay between genetic predisposition and environmental factors. Epigenetic modifications serve as a bridge between these influences, offering a mechanism through which environmental stimuli can modify gene expression and contribute to the development of epilepsy [17]. This interaction is particularly relevant in complex and multifactorial epilepsies, where genetic susceptibility alone may not fully account for disease manifestation. Moreover, epigenetic dysregulation has been implicated in various phases of epilepsy, including initiation, progression, and maintenance of seizures. DNA methylation alterations, for instance, have been associated with changes in the expression of genes crucial for synaptic plasticity, neuronal excitability, and inflammation [18-20], all pivotal players in the epileptic process. Histone modifications, on the other hand, exert nuanced control over chromatin structure, influencing the accessibility of genes to transcriptional machinery and thus modulating their expression [21, 22]. Non-coding RNAs, including microRNAs, have emerged as key players in fine-tuning gene expression post-transcriptionally. Dysregulation of specific microRNAs has been linked to aberrant expression of ion channels, neurotransmitter receptors, and other molecules implicated in epilepsy. The intricate regulatory networks orchestrated by these epigenetic mechanisms contribute significantly to the complexity and heterogeneity of epilepsy syndromes [23]. The clinical implications of understanding epigenetic regulation in epilepsy are far-reaching. Epigenetic biomarkers hold promise for improved diagnostic accuracy, prognosis, and personalized treatment strategies [24, 25]. Identifying specific epigenetic signatures associated with distinct epilepsy subtypes may pave the way for targeted therapeutic interventions, addressing the underlying molecular aberrations rather than merely managing symptoms. Furthermore, unraveling the epigenetic landscape of epilepsy opens avenues for the development of novel therapeutic approaches. Epigenetic modulators, such as DNA methyltransferase or histone deacetylase inhibitors, are under investigation for their potential antiepileptic effects. Targeting specific epigenetic pathways offers a level of precision that traditional antiepileptic drugs may lack, potentially mitigating side effects and enhancing therapeutic efficacy. The importance of epigenetic regulation in epilepsy lies in its capacity to bridge the gap between genetic predisposition and environmental influences, providing a nuanced understanding of the disease's complexity. The dynamic and reversible nature of epigenetic modifications positions them as promising targets for therapeutic interventions and underscores their significance in advancing epilepsy research and treatment paradigms. This review encompasses an examination of studies elucidating the intricate realm of epigenetic regulation in the context of epilepsy.

## 2 DNA METHYLATION IN EPIGENETICS AND ITS ROLE IN EPILEPSY

DNA methylation is currently one of the most extensively studied epigenetic mechanisms. It involves the transfer of a methyl group to the 5' carbon of cytosine in CpG dinucleotides, utilizing S-adenosylmethionine as the methyl donor through the action of DNA methyltransferases (DNMTs). The methyl group is covalently bonded to the cytosine residue in the CpG dinucleotide [26]. Genomic DNA methylation is predominantly localized in non-coding regions, such as telomeres and heterochromatin, and is also dispersed in highly repetitive sequences, such as transposons. This indicates that DNA methylation plays a crucial role in maintaining genome stability and defense mechanisms. Previously considered a stable epigenetic mark, DNA methylation is now recognized as a dynamic process, with methyl groups being added or removed from DNA residues through the activity of specific enzymes [8].

DNA methylation can modulate gene expression by interfering with the binding of sequence-specific transcription factors, thereby directly inhibiting transcription when levels are increased. Alternatively, DNA methylation can regulate gene expression through the mediation of CpG-binding proteins, such as methyl-CpG-binding protein 2 (MeCP2), which can activate or inhibit gene expression [27]. Recent research has provided evidence that DNA methylation is a significant contributor to the development and plasticity of the central nervous system, as well as neuronal repair and regeneration. Moreover, DNA methylation has been found to participate in the formation of neural functions, such as learning and memory [28].

Aberrant DNA methylation has been demonstrated to exhibit a robust association with a wide range of neurological disorders, including but not limited to epilepsy, neurodevelopmental anomalies, neurobehavioral abnormalities, neurodegenerative diseases, and central nervous system tumors [16]. This highlights the importance of utilizing DNA methylation profiling as a novel and highly valuable clinical biomarker. In a review article published in 2011, the “methylation hypothesis” was put forward for focal epilepsy, with a particular emphasis on MTLE accompanied by hippocampal sclerosis. This hypothesis posits that epileptic seizures can trigger genomic DNA methylation, causing alterations in gene expression that ultimately impact the occurrence and progression of epilepsy [29].

In 2012, Zhu *et al*. reported increased expression of DNA methyltransferase 1 and 3a (DNMT1 and DNMT3a) in the temporal neocortex of TLE patients, along with enhanced levels of DNA methylation [30]. In contrast, de Nijs *et al*. reported reduced DNA methylation levels and DNMT3a expression in the hippocampus of TLE patients in 2019 [31]. Notably, a decrease in the DNMT activity hyperhomocysteinemia-induced hypomethylation has been reported to exacerbate neuronal damage [32]. Recent research has revealed the involvement of DNA methylation in the onset and development of epilepsy. Inhibition of DNA methyltransferase in hippocampal slices or primary hippocampal neurons can reduce neuronal excitability and neural network activity. DNMTs inhibit spontaneous excitatory neurotransmitter release in the presence of MeCP proteins, while MeCP2 protein is a key regulator of gene transcription through DNA methylation [33]. Further studies have shown that abnormal DNA methylation of the RELN gene promoter in TLE patients with hippocampal sclerosis is associated with granule cell dispersion [34].

Several whole-genome studies have demonstrated changes in methylation levels during the onset and development of epilepsy [9, 35-40]. One study conducted an analysis of DNA methylation patterns in the hippocampus, amygdala, surrounding cortex to the epileptogenic zone (SCEZ), and peripheral blood of drug-resistant TLE patients using Illumina Infinium Methylation EPIC microarray technology, revealing changes in epigenetic patterns and identifying previously unreported genes associated with drug-resistant TLE, such as TBX5, EXOC7, and WRH. Moreover, CHORDC1 in peripheral blood was identified as a potential alternative biomarker [9]. Exploring peripheral blood DNA methylation changes in epilepsy is considered a promising direction for translational research, as blood samples are readily available in most clinical settings. Whole-genome DNA methylation profiling in TLE patients and then combined with selected candidate CpGs in the training cohort and validated in another independent cohort by employing machine learning algorithms, which were used to establish TLE diagnostic and drug response prediction models, and it demonstrated that DNA methylation features can define human TLE and improve the prediction of drug treatment response when combined with clinical pathological factors [41].

A prospective study was conducted to investigate the whole-genome DNA methylation changes in adult epilepsy patients receiving modified ketogenic diet therapy. The results showed a significant decrease in overall and specific site DNA methylation levels after 4 and 12 weeks of dietary treatment. A substantial proportion of identified genes were related to epilepsy and inositol metabolism, although no clear correlation with seizure severity was observed [10]. However, the study had some limitations, including the majority of patients using antiepileptic drugs, which can directly or indirectly affect DNA methylation patterns by influencing one-carbon metabolism nutrients. For example, valproic acid, one of the most commonly used antiseizure medications, has been shown to inhibit DNMT and induce a decrease in overall DNA methylation [42]. Additionally, the use of whole blood as a substitute tissue for disease manifestation in the brain is not yet fully understood. It is currently unclear how dietary-induced DNA methylation changes in blood correspond to those in the brain. However, studies have found a positive correlation between the DNA methylation patterns of peripheral tissues (including blood) and brain tissue in epilepsy patients [43].

In 2021, a study compared the DNA methylation patterns in the brains of MTLE patients and non-epileptic controls and found significant changes in the DNA methylation patterns of the hippocampus and neocortex in MTLE-HS. These changes are closely related to various biological functions, such as neuroinflammation, and have been demonstrated to be involved in the occurrence of epilepsy. Furthermore, these changes are associated with the duration of the disease in the epileptic brain [40]. In addition, DNA methylation changes can be detected in temporal lobe epilepsy animal models. By using methylation CpG capture-related large-scale parallel sequencing technology and chip-based technology to compare the whole-genome DNA methylation patterns of two TLE rodent models, it was confirmed that DNA methylation changes occur after status epilepticus or prolonged seizures and distinguish the DNA methylation characteristics of chronic epilepsy from normal conditions. Differentially methylated genes in TLE patients and animal models mainly participate in pathways or biological processes such as neuron/neuronal synapse transmission, cell survival/death, and transcription regulation [44, 45].

## 3 THE ROLE OF NON-CODING RNA IN EPILEPSY

Recent research has shown that non-coding RNA (ncRNA) plays a crucial role in both the pathological and physiological processes of the nervous system. These processes include neural development, inflammatory response, synaptic plasticity, oxidative stress, and many others [46]. This evidence suggests that ncRNA may play a significant role in the pathogenesis of epilepsy. The ncRNA refers to endogenous RNA that does not encode proteins or is not translated into proteins. Studies have indicated that only 2% of the human genome comprises protein-coding genes, while the majority of genes are primarily transcribed into ncRNA [47]. The ncRNA plays an essential role in epigenetic modifications and may have a significant impact on the molecular pathways and cellular processes of epilepsy, such as neuroinflammation, cell proliferation and differentiation, migration, apoptosis, and synaptic remodeling [48]. The ncRNA molecules are functional RNA molecules that cannot be translated into proteins and are involved in gene expression transcription and post-transcriptional regulation [49]. The main types of ncRNA include small nuclear RNA, small nucleolar RNA, microRNA (miRNA), long non-coding RNA (lncRNA), circular RNA (circRNAs), and others. Each type and subtype of ncRNA has its specific biological generation pathway, mechanism of action, and biological role [50] (Table **1**).

### 3.1 MiRNAs and Epilepsy

Neurogenesis is a crucial process in the development of epilepsy. A considerable number of miRNAs play important roles in regulating the proliferation and differentiation of various types of stem cells, suggesting their potential involvement in epilepsy [51]. In a study using a lithium-pilocarpine-induced seizure model, miR-494 was significantly downregulated, and overexpression of miR-494 inhibited receptor-interacting protein kinase 1, leading to inactivation of the NF-κB signaling pathway, accelerated cell proliferation, and inhibition of hippocampal neuron apoptosis, thereby reducing neuronal damage and inhibiting the development of epilepsy [52]. In a recent study, a combination of anti-microRNA oligonucleotides targeting miRNA-124 and miRNA-137 was injected into the hippocampus, which restored neural stem cell (NSC) proliferation and prevented the loss of NSCs in the dentate gyrus during non-convulsive seizures. Overexpression of miRNA-124 and miRNA-137 restored the changes in NSCs induced by epilepsy, indicating their synergistic role in regulating NSC proliferation and their potential in future epilepsy treatment [53].

Neurons can change their synaptic connections based on their activity, a biological process known as synaptic plasticity, which is crucial for maintaining the stability of neural pathways and the basis of memory, learning, and cognition. Increasing evidence suggests that many miRNAs are involved in the regulation of synaptic plasticity. Rajman *et al*. found that silencing miRNA-129-5p could prevent synaptic shrinkage and reduce the severity of seizures [54]. Further studies found that calcium pump Atp2b4 and doublecortin were the targets of miRNA-129-5p. Restoring the expression of Atp2b4 and doublecortin was sufficient to prevent synaptic shrinkage in neurons. As researchers continue to study miRNAs expressed in the brain, we may better understand the regulatory functions of various miRNAs in animal and human epilepsy.

Increasing evidence also reveals the role of miRNAs in regulating neuronal apoptosis. Apoptosis is a form of programmed cell death that is essential for the survival and development of organisms, controlling the activity, differentiation, and proliferation of cells. Changes in apoptotic signaling pathways have been reported in patients with TLE [55-58]. MiR-136 was significantly downregulated in the hippocampus tissue of rats with epilepsy. Overexpression of miR-136 significantly reduced the frequency and duration of seizures in rats, improved hippocampal tissue damage, and reduced neuronal damage. Overexpression of miR-136 also significantly reduced the rate of cell apoptosis in the hippocampus tissue and inhibited the level of inflammatory factors. Meanwhile, miR-136 downregulated the expression of proteins related to the Wnt/β-catenin signaling pathway. A recent study found that overexpression of miR-25-3p could downregulate oxidative stress responsive 1 (OXSR1) and reduce the occurrence of spontaneous seizures, oxidative stress response, and apoptosis. MiR-25-3p was shown to have anti-epileptic, anti-oxidative, and anti-apoptotic effects on primary cultured neurons by targeting OXSR1, providing a new target for the treatment of epilepsy [58]. In addition, recent studies have reported that miRNA-19, miR-135a-5p, miR-135b-5p, and miR-15a can participate in the process of neuronal apoptosis related to epilepsy through different molecular and cellular pathways [59-62].

The significance of inflammatory responses in the pathogenesis of epilepsy has been increasingly acknowledged [63]. miRNAs are involved in the inflammatory response during epileptogenesis. In a rat model of TLE established by intraperitoneal injection of lithium chloride-pilocarpine, there was a significant increase in the expression level of miRNA-146a in hippocampus tissue. Intraventricular injection of miRNA-146a interference RNA can reduce the expression levels of interleukin (IL)-1β, IL-6, and IL-18 in the hippocampus tissue of epileptic rats, thereby alleviating the inflammatory response [64]. Silencing miRNA-146a can alleviate oxidative stress and inflammatory response induced by epilepsy, thereby protecting against neuronal damage. Although many other miRNAs have been identified to participate in the inflammatory response associated with epilepsy, there is still insufficient direct evidence, and further research is required to clarify their mechanisms of action in the occurrence of epilepsy. RNA therapy has been highly effective in preclinical studies targeting miRNAs, and Miravirsen is the first oligonucleotide targeting miRNA tested in clinical trials for disease treatment [65]. Currently, more than a dozen different miRNAs have been inhibited by this method, and some case reports have suggested their effects on induced or spontaneous epilepsy [66]. For instance, reducing the expression of miRNA-134 in the brain using antisense oligonucleotides can increase the seizure threshold and reduce the duration of status epilepticus. Inhibiting miRNA-134 after status epilepticus can effectively reduce the occurrence of spontaneous recurrent seizures. It is noteworthy that some researchers have found abnormal expression of miRNA-134 in the plasma and cerebrospinal fluid of epilepsy patients, suggesting that miRNA-134 may serve as a diagnostic biomarker [67].

### 3.2 lncRNA and Epilepsy

lncRNAs are RNA molecules that are longer than 200 nucleotides and do not encode proteins. They have complex sequences and multiple mechanisms of action and play a critical role in controlling nuclear organization, RNA processing, transcription, and post-transcriptional gene expression regulation [68]. Some lncRNAs have been found to be involved in neuronal function, central nervous system development, and synaptic plasticity [69]. There is increasing evidence to suggest that lncRNAs play an important role in the development and progression of epilepsy.

LncRNAs are involved in the differentiation of epilepsy-related cells. LncRNA-NEAT1 is highly expressed in neurons and increases during the differentiation of neurons and glia. Studies have found that NEAT1 is significantly elevated in the cerebral cortex of epilepsy patients, particularly in high-activity areas. However, in the pilocarpine-induced epilepsy model, NEAT1 is briefly reduced during the acute phase of epilepsy. The study for this finding hypothesized that NEAT1 expression changes may vary according to temporal stimulation. NEAT1 also has the ability to regulate neuronal excitability by interacting with ion channel regulatory proteins, such as potassium channel-interacting proteins KCNAB2 and KCNIP1, which are associated with epilepsy and neuronal excitability [70]. Recently, it was found that in the developing forebrain of mice, the transcription factors regulated by lncRNA-Evf2 are crucial for the development of intermediate neurons that produce gamma-aminobutyric acid (GABA), and their absence can lead to defects in the GABAergic system. A recent study found that Evf2 can reduce susceptibility to epilepsy in a mouse model. Mice lacking Evf2 are more prone to severe and frequent seizures due to impaired GABAergic neural circuits [71].

Brain-derived neurotrophic factor (BDNF) plays a significant role in the survival of neurons during their development. It is involved in the growth and development of axons and dendrites, as well as the formation and maturation of synapses [72]. Recent studies have shown that lncRNA-PVT1 is highly expressed in the hippocampal tissue of rats with epilepsy, and silencing PVT1 improves spatial learning and memory ability, reduces neuronal loss, and decreases the expression of cleaved-caspase 3 and Bax while increasing the expression of pro-caspase-3 and Bcl-2, indicating its pivotal role in neuronal apoptosis. Silencing PVT1 also increases the expression level of BDNF in the hippocampal tissue of rats with epilepsy, suggesting that PVT1 plays a role in synaptic remodeling [73].

MALAT1 is a lncRNA that is expressed abundantly in human tissues and the cerebral cortex. It regulates many genes that are involved in dendrite and synapse development by selective splicing, transcription, and post-transcriptional regulation, which enables it to exert its molecular function [74]. In cultured hippocampal neurons, silencing MALAT1 can significantly reduce synaptic density, whereas its overexpression can increase synaptic generation. Researchers have found that silencing MALAT1 can activate the PI3K/Akt signaling pathway and protect hippocampal neurons from autophagy and apoptosis induced by epileptic rats [75]. Although MALAT1 may be a potential therapeutic target, its wide expression in the human body and involvement in many normal physiological functions, such as synaptic formation, vascular growth, and skeletal muscle development, may result in complex functional effects when only overexpressed or silenced under pathological conditions. Researchers found that the expression level of lncRNA-FTX was significantly decreased in the hippocampal tissue of the pilocarpine-induced rat model. Further research revealed that overexpression of FTX can control epileptic seizures and inhibit hippocampal neuron apoptosis. The study also uncovered a new mechanism of FTX-mediated neuronal apoptosis induced by status epilepticus through targeting the miR-21-5p/SOX7 axis. When studying the functional targets of FTX, researchers discovered that FTX can negatively regulate miR-142-5p expression during the epileptic phase by binding to its 3' untranslated region. Upregulation of miR-142-5p can inhibit iron death of rat hippocampal neurons by reducing the expression of GABPB5 [76]. These research findings suggest that FTX may provide a new target for the development of lncRNA-based therapeutic strategies.

LncRNAs have been shown to play a crucial role in the inflammatory response of MTLE. Specifically, lncRNA-H19 has been found to be involved in the inflammatory response of epilepsy. Overexpression of H19 can activate microglia and astrocytes by regulating the JAK/STAT pathway, which in turn stimulates the release of pro-inflammatory cytokines (IL-1B, IL-6, and TNF-x) in the hippocampus. Conversely, silencing H19 can inhibit the activation of neuroglial cells induced by epilepsy and reduce the inflammatory response, highlighting its important role in the inflammatory response [77]. Another lncRNA, lncRNA-NEAT1, has been shown to have significantly elevated levels in hippocampal neurons of epilepsy patients. Silencing NEAT1 has been found to significantly reduce the mRNA expression levels of pro-inflammatory cytokines (IL-6, cox-2, and TNF-α). Further research has revealed that lncRNA NEAT1 can affect the inflammatory response and cell vitality in epilepsy by targeting miRNA-129-5p and further regulating the Notch signaling pathway [78]. LncRNA-UCA1 has also been shown to play a role in the pathogenesis of epilepsy by regulating miRNA-203 to promote the expression of myocyte enhancer factor 2C and inhibit the NF-xB signaling pathway [79]. In animal experiments, PVT1 has been found to promote neuronal cell damage and neuroinflammation induced by epilepsy by regulating the FOXD3/SCN2A pathway mediated by miR-488-3p [80]. Above findings have shed light on the mechanism of epileptic inflammatory response and provided promising therapeutic strategies for epilepsy. For instance, oligonucleotides targeting long non-coding RNA-SCN1ANAT have been found to increase the mRNA level of SCN1A in cell models and prevent seizures caused by high body temperature in Dravet mouse model. This treatment has been introduced into clinical trials for Dravet syndrome [81]. However, the fact that large oligonucleotides cannot pass through the blood-brain barrier means that therapeutic drugs must bypass this problem. In summary, RNA-based therapy provides the ability to selectively manipulate many genes, and as researchers continue to discover the role of non-coding RNA in the future, there may be more opportunities to develop new treatment methods for epilepsy.

### 3.3 Circular RNAs and Epilepsy

CircRNAs are a recently discovered class of endogenous non-coding RNAs characterized by their highly stable, covalently closed structure. Studies have revealed that their significant enrichment in the nervous system [82]. Notably, circRNAs play important roles in key neurobiological processes, including neurotransmitter-related tasks, neuronal maturation, and synaptic function. Furthermore, they have been associated with host genes in pathways such as axon guidance, Wnt signaling, and transforming growth factor-β signaling, which is known to participate in substantive functions, such as neuronal migration and differentiation, as well as axonal specificity, and play a role in brain development [83].

While the precise mechanisms of action of circRNAs are still being elucidated, emerging research suggests that they can function as miRNA sponges or molecular scaffolds for RNA-binding proteins in some species. Additionally, circRNAs have demonstrated the ability to control the transcription of their host genes and even directly synthesize short peptides/proteins [84]. This multifaceted nature of circRNAs highlights their potential significance in various biological processes, including those related to epilepsy. In the context of epilepsy research, a study utilizing second-generation sequencing was conducted to sequence the hippocampal tissue of epileptic mice; this study revealed differential expression of 100 circRNAs. Subsequently, bioinformatics analysis was employed to construct an abnormal circRNA-miRNA-mRNA regulatory network. Gene ontology and pathway analysis indicated that the abnormal mRNAs in these networks were closely associated with the fundamental processes of epilepsy. Abnormal circRNAs may thus regulate epilepsy-related mRNAs *via* circRNA-miRNA-mRNA interaction, thereby playing a pathophysiological role in chronic epilepsy [85].

Another study utilized gene chips to identify 586 circRNAs with differential expression in the brain tissue of TLE patients. Of note, CircRNA-0067835 was found to be significantly abnormally expressed in both epileptic tissue and plasma. Lower circRNA-0067835 expression was associated with higher seizure frequency, hippocampal sclerosis, and Engel scores. Overexpression of circRNA-0067835 significantly inhibited the proliferation of SH-SY5Y cells, caused G1 phase arrest, and promoted cell apoptosis. Further research revealed that circRNA-0067835 acted as a miRNA-155 sponge to promote the expression of FOXO3a, thereby affecting the progression of intractable epilepsy. This suggests that circRNA-0067835 may be a potential therapeutic target for TLE patients [86]. Recently, it was found that HsTx regulated the circ_0001293/miR-8114/TGF-β2 axis mainly through anti-inflammatory activity, significantly preventing the progression of epilepsy [87].

While circRNAs have not yet been established as a biomarker for epilepsy, their structural stability and abundant expression suggest the possibility of further research uncovering effective biomarkers for epilepsy or other neurological diseases. In conclusion, circRNA, as a newly discovered non-coding RNAs, are gaining recognition for their roles in human diseases, but their specific contributions to the molecular pathways and mechanisms of epilepsy require further investigation.

## 4 THE ROLE OF HISTONE MODIFICATIONS IN EPILEPSY

Histones are highly conserved structural proteins that play a fundamental role in the formation of eukaryotic chromosomes. There are five main types of histones: H1, H2A, H2B, H3, and H4. Histone modification is a crucial form of epigenetic regulation that can alter the affinity of histones for DNA or other proteins, thereby playing a critical role in chromatin structure and function. The impact of single or multiple histone modifications on transcription is complex. The N-terminal tails of histones are subject to a wide range of modifications, including acetylation, methylation, phosphorylation, ubiquitination, and more [88]. Subsequently, proteins with specific domains recognize these chemical modifications and recruit transcriptional activators or repressors to specific modification sites to regulate gene expression. In recent years, the relationship between histone modifications and epilepsy has become a popular research topic, with extensive studies conducted in various epilepsy models or patients. Histone modifications not only play a crucial role in the normal development of the central nervous system but also participate in the occurrence and development of epilepsy-mediated gene regulation [22, 89].

### 4.1 Histone Acetylation

Histone acetylation is catalyzed by histone acetyltransferases (HATs), which induce chromatin relaxation and facilitate the binding of transcription factors to specific sites, ultimately activating gene expression. In contrast, histone deacetylation is catalyzed by histone deacetylases (HDACs), which remove acetyl groups from core histones, leading to chromatin compaction and inhibition of gene transcription [22]. The dynamic regulation of histone acetylation by HATs and HDACs is crucial for proper neuronal function, structural plasticity, and gene expression. Acetylation modifications participate in numerous neurobiological processes, such as neuronal death, glial cell proliferation, neuroinflammation, ion channels and neurotransmitter receptors changes, axonal and dendritic plasticity, and the neural network remodeling involved in the occurrence and development of epilepsy [90, 91]. Histone acetylation levels have been observed to change in epilepsy animal models. For example, Huang *et al*. and Tsankova *et al*. reported that the levels of histone H4 acetylation were enhanced in the pentylenetetrazol and electrically induced epilepsy models [92, 93]. Additionally, Crosio *et al*. reported a significant increase in the levels of histone H3 phosphorylation in the pilocarpine and kainic acid-induced epilepsy models [94]. Sng *et al*. further confirmed these findings and demonstrated that histone H3 phosphorylation was restricted to dentate gyrus neurons, where it rapidly increased and then decreased, while histone H4 acetylation appeared later and was expressed in most hippocampal tissues [95]. This study also revealed that histone H4 acetylation was associated with the induction of the oncogene c-fos [95]. Studies have shown that the expression of HDACs, such as HDAC2 and HDAC1, is upregulated in patients with MTLE and animal models [11, 96, 97]. Changes in HDAC expression can affect the transcriptional response of certain genes, such as ionotropic glutamate receptors and metabotropic glutamate receptors [98].

In an animal model of epilepsy, pretreatment with the HDAC inhibitor curcumin in mice reduced the levels of histone modifications and blocked c-fos induction after seizure, suggesting that histone modifications can directly affect gene expression after seizure [95]. Although HDACs have been found to have potential roles in epilepsy, only a few studies have explored their roles in the development of epilepsy, most of which were conducted in animal models. Histone deacetylase inhibitors have a long history of use in treating various neurological diseases, including epilepsy [99]. Histone deacetylase inhibition has been shown to prevent the development and persistence of TLE in animal models [89].

### 4.2 Methylation of Histones

Methylation of histones, particularly at the N-terminal and C-terminal ends of lysine and arginine residues, represents a crucial modification that can either repress or enhance gene expression. This modification plays a significant role in regulating transcription at various stages, mRNA splicing, DNA repair, and replication. The control of histone methylation is dependent on the activity of histone methyltransferases and histone demethylases. Histone methylation can have either an inhibitory or activating effect on transcription, depending on the number and site of methylation. Histone methyltransferases play a pivotal role in brain development and brain diseases, and changes in their activity can lead to intellectual disability with epilepsy syndrome [100]. Kleefstra syndrome is a disorder characterized by severe mental retardation, seizures, and behavioral abnormalities and is often linked to EHMT1 gene mutations [101]. EHMT1 protein, a histone methyltransferase, is implicated in learning and memory. Patients with Kleefstra syndrome who test negative for EHMT1 gene mutations may also have mutations in other epigenetic regulatory factors, such as lysine-specific methyltransferase like KMT2C [102].

Histone lysine demethylase 5C (KDM5C), a lysine-specific demethylase, has an unknown function in the central nervous system, and mutations in it may contribute to X-linked intellectual disability [103]. Mutations in the aristaless-related homeobox gene are associated with early infantile epileptic encephalopathy (also known as Ohtahara syndrome) and other epileptic encephalopathies such as West syndrome and X-linked myoclonic epilepsy [104]. ARX protein is also a significant regulatory factor for KDM5C enzyme and indirectly affects histone methylation [105]. Histone methyltransferase nuclear receptor-binding SET domain protein 1 is associated with various overgrowth phenotypes, including some cases of Beckwith-Wiedemann syndrome, Weaver syndrome, and most cases of Sotos syndrome [106]. A considerable decrease in the relative abundance of H3K9me2 and G9a is evident in the promoter region of the Kcnj10 gene within the epileptic hippocampus. The removal of the repressive mark H3K9me2 is linked to a reduction in transcriptional inhibition of the Kcnj10 gene, thereby leading to its increased expression. The study at hand presents compelling evidence that H3K9me2 and G9a are responsive to epileptic seizure activity and can significantly modulate the transcriptional regulation of the Kcnj10 channel during the acute phase of epilepsy [107].

## 5 OTHER EPIGENETIC MECHANISMS

In addition to DNA methylation, histone modifications, and non-coding RNA, RNA itself can undergo a variety of modifications. As a post-transcriptional modification, RNA methylation is an important epigenetic mechanism. Two types of RNA methylation, 5-methylcytosine (5-mC) and N6-methyladenosine (m6A), are involved in the methylation of various RNA species, including messenger RNA and non-coding RNA. Studies have shown that RNA 5-mC and m6A methylation play critical roles in the regulation of diverse biological processes such as RNA stability and mRNA translation [108]. FTO, an endogenous enzyme responsible for m6A demethylation in RNA, is highly expressed in brain tissue and is closely related to nervous system development [109]. Non-synonymous mutations in the FTO enzyme domain have been linked to brain developmental abnormalities and neurological dysfunction. In addition, m6A methylation can regulate ncRNA, and some ncRNA species have been implicated in epilepsy, suggesting that abnormal RNA modification may be involved in the pathogenesis of this disorder.

## 6 SUMMARY AND FUTURE DIRECTIONS

To determine the causality of epigenetic modifications in the development of epilepsy or whether they are a consequence of recurrent seizures, it is crucial to examine the time point shortly after the first status event, like status epilepticus trauma, febrile seizures, and encephalitis, status epilepticus is an initial damaging event in many TLE models, characterized by prolonged convulsive seizures. After the initial seizure, both humans and rodents enter a latent period, during which they do not experience seizures before developing epilepsy. However, not all patients progress to epilepsy after their first seizure. The latent period between status epilepticus and recurrent seizures is essential for understanding the brain connectivity changes in epilepsy. Epigenetics has become increasingly important at this critical time point before epilepsy onset. Epigenetic changes visible between status epilepticus and long-term epilepsy may play a role in switching to an epileptic brain state, including inflammatory responses, cell apoptosis, synaptic remodeling, and cell differentiation. Epigenetic studies in epilepsy are still in their nascent stages, and the tissue and time specificity of epigenetic modifications, as well as the relationship between epigenetic changes and the occurrence and development of epilepsy, require further elucidation. Analyzing epigenetic changes in epilepsy is a formidable and intricate undertaking, but it holds significant implications. In the future, drugs that specifically target DNMTs and histone lysine deacetylase may provide novel therapeutic options for patients with epilepsy who are refractory to currently available antiepileptic drugs. Research into the relationship between histone demethylation and epilepsy is still in its infancy; nonetheless, studies have suggested that histone demethylation can modulate neuronal excitability and synaptic plasticity, leading to alterations in brain network function and structure that promote the development and progression of epilepsy. Current clinical approaches for treating epilepsy are limited in terms of efficacy and are associated with varying side effects. Histone modifications have emerged as a focus of research in understanding the pathogenesis of epilepsy. Further exploration of the correlation between epigenetic modifications, particularly histone modifications, and epilepsy is essential to gain novel insights into the pathogenesis of epilepsy and new therapeutic targets. Drugs that modulate the epigenetic machinery are currently available and under development for a wide range of human diseases, and there is evidence to suggest that some commonly used antiepileptic drugs may partially exert their therapeutic effects through epigenetic mechanisms. Most research into epigenetic changes in epilepsy has focused on DNA methylation. However, recent studies have generated significant interest in the role of microRNAs as potential biomarkers for early clinical diagnosis and prognosis. Non-coding RNAs may represent a promising therapeutic target for epilepsy due to their high selectivity, which can be achieved using antisense nucleic acid-based oligonucleotide inhibitors. Advanced gene editing techniques and non-coding RNA regulation hold promise for enabling early diagnosis and targeted treatment of epilepsy. Moreover, compared to coding RNA, our understanding of non-coding RNA remains limited, and many of their functions remain unclear. Therefore, further research into the mechanisms underlying non-coding RNA action may lead to a deeper understanding of the pathogenesis of epilepsy, its development, and progression. Enhancing our understanding of the critical role of epigenetics in the pathogenesis of epilepsy may not only deepen our understanding of the disease but also aid in the identification of novel biomarkers and therapeutic targets for epilepsy. Epigenetic modifications may represent a direct target for epilepsy treatment and may, therefore, open up new avenues for the development of antiepileptic drugs. With the development and refinement of various high-throughput gene sequencing platforms, epigenomic data at different levels are emerging. Multidimensional exploration and integration of the biological mechanisms and clinical translation of this big data pose significant challenges.

## Figures and Tables

**Table 1:** The role of non-coding RNA in epilepsy.

**Non-coding RNA**	**Source**	**Role in Epilepsy**	**References**
miR-494	lithium-pilocarpine-induced epilepsy model	Elevated miR-494 repressed RIPK1, causing an inactivation of the NF-κB signaling pathway and acceleration of cell proliferation, and suppression of apoptosis of hippocampal neurons in Ep rats, thereby attenuating the neuron injury and Ep development.	[42]
miR-129-5p	Hippocampal electrode implantation in rat models, kainic acid-induced epileptic rat models, and primary culture of hippocampal cells	Silencing miR-129-5p prevents synaptic shrinkage and reduces the severity of epileptic seizures; calcium pump Atp2b4 and doublecortin are targets of miR-129-5p.	[54]
miR-25-3p	Models of hippocampal cells in magnesium-free culture medium and kainic acid-induced epileptic rat models	miR-25-3p exerts antiepileptic, antioxidant, and anti-apoptotic effects on primary cultured neurons by targeting OxSR1.	[58]
miR-146a	Hippocampal tissue from pilocarpine-induced epileptic rats	Silencing microRNA-146a alleviates neurodamage in temporal lobe epilepsy.	[64]
miR-134	Adult temporal lobe epilepsy specimens, kainic acid-induced epileptic mice and rats models	miRNA-134 restricts the working range of neurons by altering dendritic spines.	[67]
lncRNA-NEAT1	Adult temporal lobe epilepsy specimens and CTX-TNA astrocyte cell line	Inhibition of miRNA-129-5p and further modulation of the Notch signaling pathway affect inflammation response and cell vitality; interaction with epilepsy-associated potassium channel interacting proteins (including KCNAB2 and KCNIP1) regulates neuronal excitability.	[70]
lncRNA-Evf2	Gene knockout mouse models	Transcription factors play a crucial role in the development of GABA-producing interneurons.	[71]
lncRNA-PVT1	Hippocampal tissue from pilocarpine-induced epileptic rat models	Inhibiting PVT1 downregulates the Wnt signaling pathway, reduces neuronal loss, suppresses astrocyte activation, and increases hippocampal BDNF expression.	[73]
lncRNA-FTX	Pilocarpine-induced epileptic rat models and primary culture of hippocampal cells	FTX regulates apoptosis in SE-induced neurodamage through the miR-21-5p/SOX7 axis.	[76]
lncRNA-H19	Adult temporal lobe epilepsy specimens, pilocarpine-induced and kainic acid-induced epileptic rat hippocampal tissue	H19 activates the JAK/STAT signaling pathway by promoting Stat3 and c-Myc expression; H19 may act as an endogenous competing RNA, sequestering let-7b to participate in the regulation of apoptosis.	[77]
lncRNA-UCA1	Hippocampal tissue from pilocarpine-induced epileptic rat models and *in vitro* models	lncRNA UCA1 inhibits inflammation and epilepsy by suppressing the mir-203/MEF2C/NF-κB axis; UCA1 inhibits hippocampal neuron apoptosis through the miR-495/Nrf2-ARE pathway.	[79]
circRNA-0067835	Brain tissue from temporal lobe epilepsy patients and SH-SY5Y cells	CircRNA-0067835 acts as a sponge for miRNA-155, promoting FOXO3a expression and thus affecting the progression of refractory epilepsy.	[86]
circ_0001293	Pentylenetetrazol-induced epilepsy model	HsTx regulated the circ_0001293/miR-8114/TGF-β2 axis mainly through anti-inflammatory activity, significantly preventing the progression of epilepsy.	[87]

## LIST OF ABBREVIATIONS

5-mC: 5-methylcytosine
BDNF: Brain-derived Neurotrophic Factor
circRNAs: Circular RNA
DNMT1: DNA Methyltransferase 1
DNMT3a: DNA Methyltransferase 3a
DNMTs: DNA Methyltransferases
GABA: Gamma-aminobutyric Acid
HATs: Histone Acetyltransferases
HDACs: Histone Deacetylases
IL: Interleukin
KDM5C: Histone Lysine Demethylase 5C
lncRNA: Long Non-coding RNA
MeCP2: Methyl-CpG-binding Protein 2
miRNA: MicroRNA
MTLE: Mesial Temporal Lobe Epilepsy
ncRNA: Non-coding RNA
NSC: Neural Stem Cell
OXSR1: Oxidative Stress Responsive 1
snRNA: Small Nuclear RNA
TLE: Temporal Lobe Epilepsy
